# Relationship between psychological resilience, cognitive flexibility and post-traumatic growth level in patients with severe sepsis treated by continuous renal replacement therapy

**DOI:** 10.3389/fpubh.2025.1589223

**Published:** 2025-05-07

**Authors:** Changzheng Zhao, Lancai Zhou, Haixia Gao, Yongzan Lu, MiaoMiao Shi

**Affiliations:** ^1^Intensive Care Unit, Shandong Public Health Clinical Center, Jinan, Shandong, China; ^2^Surgical Intensive Care Unit, Shandong Public Health Clinical Center, Jinan, Shandong, China

**Keywords:** severe sepsis, continuous renal replacement therapy, psychological resilience, cognitive flexibility, post-traumatic growth

## Abstract

**Objective:**

Continuous renal replacement therapy (CRRT) is the primary treatment for severe sepsis and has been shown to reduce patient mortality. Patients with severe sepsis who receive CRRT frequently experience significant physical and psychological distress, manifesting as shame, social withdrawal, and abnormal cognitive moods. This study aimed to explore the relationship between psychological resilience, cognitive flexibility, and post-traumatic growth (PTG) levels in patients with severe sepsis treated with CRRT.

**Methods:**

From January to October 2024, patients with severe sepsis who were treated with CRRT in our hospital were selected by convenience sampling as the research object. The Connor-Davidson Resilience Scale (CDRISC), Cognitive Flexibility Scale, and Post-Traumatic Growth Inventory (PTGI) were used to evaluate patients’ psychological resilience, cognitive flexibility, and PTG levels. Statistical methods included the independent sample t test, Pearson analysis, and linear regression analysis.

**Results:**

The total scores of CDRISC, cognitive flexibility scale and PTGI in 205 patients was (42.98 ± 6.13), (95.04 ± 17.98) and (49.77 ± 9.92), respectively. There was a significant positive correlation between psychological resilience, cognitive flexibility, and PTG levels in patients with severe sepsis treated with CRRT (*p* < 0.05). Psychological resilience and cognitive flexibility had positive predictive effects on PTG, and there were significant positive predictive effects between psychological resilience and cognitive flexibility (*p* < 0.05). Psychological resilience directly and positively predicted PTG (*β* = 0.538, *p* < 0.05). The indirect effect of psychological resilience on cognitive flexibility was significant (*β* = 0.677, *p* < 0.05), and the indirect effect of cognitive flexibility on PTG was significant (*β* = 0.165, *p* < 0.05). The chain-mediating effect between psychological resilience, cognitive flexibility, and PTG was significant (*β* = 0.112, *p* < 0.05).

**Conclusion:**

Psychological resilience can affect the PTG level of patients with severe sepsis treated with CRRT and can also indirectly affect PTG levels through direct chain mediation of cognitive flexibility. Targeted intervention strategies should be formulated to improve mental health and promote clinical prognosis.

## 1 Introduction

Severe sepsis is a systemic inflammatory response syndrome caused by pathogens invading the blood circulation and is one of the main diseases leading to the death of intensive care patients (1). Continuous renal replacement therapy (CRRT) is widely used in the clinical treatment of severe sepsis as it effectively eliminates inflammatory factors and metabolic waste from the blood while replacing compromised renal function (2). However, long-term clinical observations have revealed that patients with severe sepsis undergoing CRRT often experience significant physical and psychological distress, frequently manifesting issues such as shame, social withdrawal, and cognitive-emotional abnormalities (3).

Resilience, also termed psychological resilience, refers to the developmental phenomenon wherein individuals maintain or restore adaptive functioning following adversity or traumatic experiences (4). Psychological resilience can improve one’s ability to perform daily activities and reduce the adverse consequences of physical dysfunction. Empirical evidence shows a correlation between post-traumatic growth (PTG) and psychological resilience in gynecological cancer patients, pneumonia survivors, and accidental trauma populations (5, 6). However, there is a lack of research to confirm this relationship in patients with severe sepsis.

Cognitive flexibility is the ability of individuals to adjust their cognition flexibly to adapt to the environment in new situations (7). Empirical investigations have established a statistically significant positive correlation between psychological and cognitive flexibility. The development of cognitive flexibility can enhance an individual’s psychological resilience and promote confidence in the face of stress, frustration, and stress. At the same time, some scholars have proposed that the relationship between psychological resilience and PTG can be explained by cognitive flexibility, but the path is not clear (8).

Post-traumatic growth denotes the positive psychological changes that occur when an individual experiences trauma (9). Clinical evidence demonstrates that PTG facilitates the establishment of adaptive self-perception and optimal psychological functioning in patients, thereby improving clinical prognosis (10). Therefore, it is important to study the level of PTG and its related influencing factors in patients with severe sepsis who receive CRRT to improve their psychological state. At the same time, some scholars have proposed that the relationship between psychological resilience and PTG can be explained by cognitive flexibility, but the underlying pathways remain insufficiently elucidated (11, 12).

This study aimed to investigate the pathways through which psychological resilience and cognitive flexibility influence post-traumatic growth in patients with severe sepsis undergoing CRRT, with the goal of providing a scientific foundation for the development of targeted psychological training programs in clinical practice.

## 2 Materials and methods

### 2.1 Subjects

From January to October 2024, patients with severe sepsis who underwent CRRT in the Department of Nephrology of our hospital were recruited using convenience sampling. The inclusion criteria were as follows: patients diagnosed with severe sepsis based on clinical manifestations and auxiliary examinations, including imaging and pathology; patients who received CRRT; those with complete clinical data; and patients with sufficient listening, speaking, reading, and writing abilities to complete the study questionnaire during hospitalization. The exclusion criteria included patients with cognitive impairment, those who had previously participated in psychological counseling or other scientific research projects, patients with other serious physical illnesses, and those with a history of mental disorders, such as anxiety or depression, prior to study enrollment.

### 2.2 Assessment tools

The following assessment tools were used in this study. First, a general information questionnaire was administered to collect demographic and clinical characteristics including age, sex, education level, marital status, monthly household income, payment method, body mass index (BMI), and etiology.

Second, psychological resilience was evaluated using the Connor-Davidson Resilience Scale (CD-RISC), which comprises 25 items across three dimensions: optimism (4 items), strength (8 items), and resilience (13 items). The optimism dimension assesses positive attitudes and future expectations; the strength dimension measures self-confidence and perceived self-efficacy; and the resilience dimension evaluates the ability to cope with adversity and stress. Each item is rated on a 5-point Likert scale (0 = never, 1 = rarely, 2 = sometimes, 3 = often, and 4 = almost always). For example, item 1, “I am able to adapt to change,” is scored from 0 to 4 based on the respondent’s selection. The total score ranges from 0 to 100, with higher scores indicating greater psychological resilience (13). In this study, Cronbach’s *α* coefficient for the CD-RISC was 0.912, indicating excellent internal consistency.

Third, cognitive flexibility was assessed using the Cognitive Flexibility Scale, which contains 20 items grouped into two dimensions: choice (13 items) and control (7 items). Responses are rated on a 5-point Likert scale (1 = never to 5 = always). Items 2, 4, 7, 9, 11, and 17 are reverse scored. Higher total scores reflect greater cognitive flexibility (14). The scale demonstrated good reliability in this study, with a Cronbach’s *α* coefficient of 0.896.

Lastly, PTG was measured using the Post-Traumatic Growth Inventory (PTGI), which includes 21 items across five dimensions: appreciation of life (six items), personal strength (three items), relating to others (three items), self-transformation (four items), and new possibilities (four items). Each item is rated on a 6-point Likert scale. PTG was present if the total score was ≥63 (15). The PTGI showed acceptable internal consistency in this study, with a Cronbach’s *α* coefficient of 0.831.

### 2.3 Quality control

All questionnaires were distributed and recovered by the researchers. Before filling in, the purpose of this study was explained to the patients, and the instructions were uniformly accepted during the filling process. All questionnaires were collected on the spot, 218 questionnaires were distributed, 13 invalid questionnaires were eliminated, and 205 questionnaires were collected.

### 2.4 Statistical methods

SPSS 24.0 was used to conduct the common method deviation test, descriptive statistical analysis, and correlation analysis. Counting data are expressed as frequency and rate (%). The measurement data were tested using the Shapiro–Wilk normal distribution, which conforms to the normal distribution and is expressed as the mean ± standard deviation ( $\bar{x}$ ±s). Pearson correlation and multiple linear regression analyses were used for regression analysis. PROCESS was used to test the mediating effect, and the Harman single-factor test was used to check the degree of deviation of the common method in this study. The significance of mediation was tested using the nonparametric percentile bootstrap method. Sampling was repeated 5,000 times, and a confidence interval of 95% was estimated. If the confidence interval did not contain 0, then the mediation effect was significant.

## 3 Results

### 3.1 General information of the research object

A total of 218 questionnaires were sent, and 205 valid questionnaires were returned, with an effective recovery rate of 94.04%. The general characteristics of the 205 subjects are presented in Table 1.

**Table 1:** General information.

General information		$\bar{x}$ ±S/n	Proportion (%)
Age (year)		50.43 ± 8.43	-
Gender	Male	114	55.61
	Female	91	44.39
Education degree	Primary school or below	39	19.02
	Junior school, high school and technical secondary school	74	36.10
	College or undergraduate	65	31.71
	Graduate and above	27	13.17
Marital status	Married	142	69.27
	Single	63	30.73
Family monthly income (yuan)	<1,000	24	11.71
	1,000 ~ 3,000	36	17.56
	3,000 ~ 5,000	90	43.90
	>5,000	55	26.83
Types of insurance	Self-paid	26	12.68
	Rural cooperative medical care	73	35.61
	Employee insurance	106	51.71
BMI (kg/㎡)		23.95 ± 2.47	-
Etiology	Pneumonia	59	28.78
	Pancreatitis	53	25.85
	Biliary tract infection	71	34.63
	Others	22	10.73

### 3.2 Psychological resilience, cognitive flexibility, and PTG level of patients with severe sepsis treated by CRRT

The total score of CDRISC, cognitive flexibility scale, and PTGI in 205 patients was (42.98 ± 6.13), (95.04 ± 17.98) and (49.77 ± 9.92), respectively. The scores for the remaining dimensions are presented in Table 2.

**Table 2:** The scores of CDRISC, cognitive flexibility scale, and PTGI in 205 patients ( $\bar{x}$ ±s, points).

Scale	Item	Score
CDRISC	Optimism	22.74 ± 3.52
	Strength	14.75 ± 1.47
	Flexibility	5.49 ± 1.14
Cognitive flexibility scale	Choice	60.40 ± 9.67
	Control	34.64 ± 8.31
PTGI	Perception of life	15.11 ± 4.25
	Personal strength	9.24 ± 1.08
	Relationship with others	8.96 ± 1.52
	Self-transformation	9.64 ± 1.46
	New possibilities	6.81 ± 1.61

### 3.3 Correlation analysis of psychological resilience, cognitive flexibility, and PTG level

There was a significant positive correlation between psychological resilience, cognitive flexibility, and PTG levels in patients with severe sepsis treated with CRRT (*p* < 0.05). The correlations of the dimensions of psychological resilience, cognitive flexibility, and the PTG scale are shown in Table 3.

**Table 3:** Correlation analysis of each variable scale.

Variable		1	2	3	4	5	6	7	8	9	10	11	12	13
CDRISC	Optimism (1)	1												
	Strength (2)	0.469^**^	1											
	Flexibility (3)	0.233^**^	0.317^**^	1										
	Total score (4)	0.890^**^	0.781^**^	0.489^**^	1									
Psychological resilience	Choice (5)	0.136	0.362^**^	0.117	0.261^**^	1								
	Control (6)	0.194^**^	0.275^**^	0.173^*^	0.275^**^	0.202^**^	1							
	Total score (7)	0.216^**^	0.403^**^	0.190^**^	0.345^**^	0.717^**^	0.828^**^	1						
	Perception of life (8)	−0.030	0.039	−0.006	−0.005	−0.055	−0.016	−0.043	1					
PTGI	Personal strength (9)	0.141^*^	0.127	0.160^*^	0.177^*^	−0.047	0.029	−0.007	0.037	1				
	Relationship with others (10)	−0.058	0.006	0.022	−0.030	0.085	0.090	0.113	−0.007	−0.071	1			
	Self-transformation (11)	0.019	0.053	−0.007	0.032	0.012	−0.096	−0.062	0.103	−0.015	0.038	1		
	New possibility (12)	0.063	0.057	−0.069	0.050	0.012	0.142^*^	0.108	0.094	0.059	0.069	−0.033	1	
	Total score (13)	0.029	0.102	0.018	0.064	−0.011	0.011	0.001	0.694^**^	0.258^**^	0.299^**^	0.605^**^	0.36^1**^	1

^*^p < 0.05, ^**^p < 0.01.

### 3.4 Analysis of the mediating effect of psychological resilience, cognitive flexibility, and PTG

Regression analysis revealed that psychological resilience and cognitive flexibility significantly and positively predicted PTG. Additionally, psychological resilience was found to have a significant positive predictive effect on cognitive flexibility. Both psychological and cognitive resilience exhibited significant direct effects on PTG. Furthermore, cognitive flexibility partially mediated the relationship between psychological resilience and PTG (Table 4).

**Table 4:** Regression analysis of the variable relationships in the mediation model.

Item	Cognitive flexibility					PTG				
	*B*	*SE*	*t*	*p*	*β*	*B*	*SE*	*t*	*p*	*β*
Constant	70.967	5.603	12.665	0.000	-	24.780	3.588	6.906	0.000	-
Psychological resilience	0.677	0.129	5.242	0.000	0.345	0.538	0.066	8.169	0.000	0.474
Cognitive flexibility	-	-	-	-	-	0.165	0.034	4.919	0.000	0.286
*R* ^2^	0.119					0.400				
*F/p*	*F* (1,203) = 27.481, *p* = 0.000					*F* (2,202) = 67.371, *P* = 0.000				

### 3.5 Mediating effect analysis

**Figure 1:**
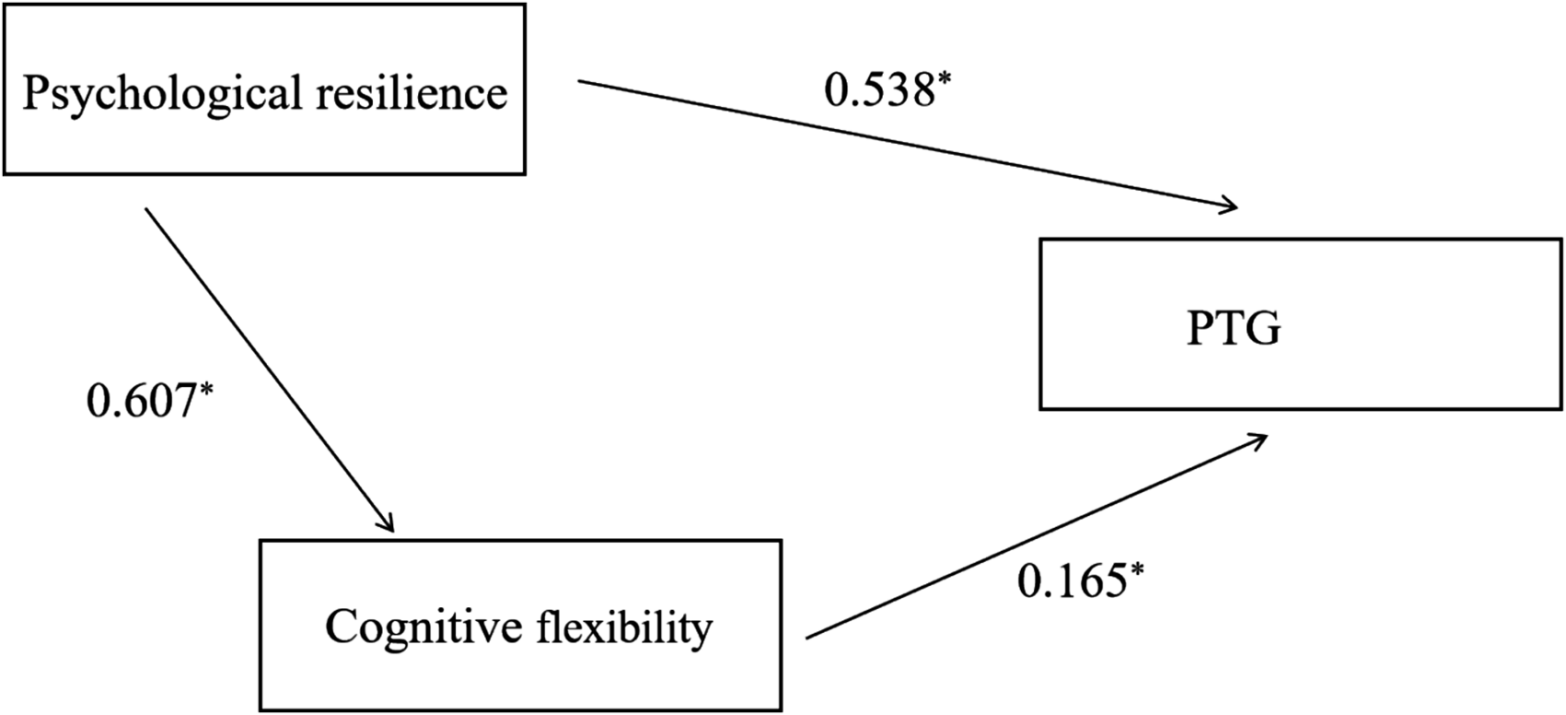
Path diagram of mediating effect of psychological resilience, cognitive flexibility, and PTG in patients with severe sepsis treated by CRRT.

Psychological resilience demonstrated a significant direct effect on PTG (*β* = 0.538, *p* < 0.001), with a 95% confidence interval (CI) of (0.409, 0.667). An indirect effect was identified through the mediating pathway: psychological resilience → cognitive flexibility → PTG. The size of the indirect effect was calculated as 0.677 × 0.165 = 0.112. A bootstrap test further confirmed the significance of this mediating effect, with a 95% CI of (0.047, 0.159). The indirect effect accounted for 17.3% of the total effect of psychological resilience on PTG. The total effect of psychological resilience on PTG was also statistically significant (*β* = 0.649, *p* < 0.001), with a 95% CI of (0.522, 0.777) (Table 5 and Figure 1).

**Table 5:** Analysis of the mediation effects.

Model effect	Item	Effect	Boot *SE*	*t*	*p*	BootLLCI	BootULCI
Direct effect	Psychological resilience⇒PTG	0.538	0.066	8.169	0.000	0.409	0.667
Indirect effect	Psychological resilience⇒Cognitive flexibility	0.677	0.129	5.242	0.000	0.424	0.930
	Cognitive flexibility⇒PTG	0.165	0.034	4.919	0.000	0.099	0.231
	Psychological resilience⇒Cognitive flexibility⇒PTG	0.112	0.028	3.925	0.000	0.047	0.159
Total effect	Psychological resilience⇒PTG	0.649	0.065	9.96	0.000	0.522	0.777

## 4 Discussion

Sepsis is a significant public health concern in China. In recent years, with the advancement of the positive psychology movement, increasing attention has been directed toward fostering positive mental health in clinical settings (16, 17). In this study, the mean total scores of CD-RISC, the Cognitive Flexibility Scale, and PTGI among 205 patients with severe sepsis undergoing CRRT were 42.98 ± 6.13, 95.04 ± 17.98, and 49.77 ± 9.92, respectively. These scores approximated a median value of 50, indicating a moderate level. This finding aligns with those of previous studies conducted in critically ill populations, including patients undergoing kidney transplantation (18, 19). These results suggest that psychological resilience, cognitive flexibility, and PTG levels in patients with severe sepsis receiving CRRT remain suboptimal and warrant further enhancement.

A plausible explanation for these findings is that severe sepsis is commonly associated with intense physical suffering, high mortality rates, and prolonged medical expenses. These stressors create uncertainty regarding disease prognosis and future life outcomes, which may hinder the development of positive self-perception and thus negatively affect psychological resilience, cognitive flexibility, and PTG levels (20).

This study further explored the mediating and interrelated roles of psychological resilience, cognitive flexibility, and PTG in patients with severe sepsis who underwent CRRT. The findings revealed that psychological resilience and cognitive flexibility significantly and positively predicted PTG. Moreover, a mutually reinforcing relationship was observed between psychological and cognitive flexibilities. Higher levels of these traits were associated with enhanced PTG, which is consistent with previous research (21). This finding supports the conclusion that both psychological resilience and cognitive flexibility are critical contributors to PTG. Enhancing these traits may facilitate the recovery and psychological growth of patients with severe sepsis. Additionally, the results demonstrated that psychological resilience and cognitive flexibility exerted significant chain mediation effects on PTG. These effects were evident through three indirect pathways: the independent effects of psychological resilience and cognitive flexibility as well as their combined mediating role.

A deeper analysis revealed that PTG in patients with severe sepsis reflects their cognitive and emotional restructuring when confronted with life-threatening illness (22). Psychological resilience plays a pivotal role in this process, enabling patients to maintain a proactive outlook on illness management and prognosis, thereby exerting a strong positive influence on PTG (23–25). Furthermore, individuals with high psychological resilience are more likely to adopt adaptive and flexible thinking patterns, thus enhancing cognitive flexibility and reducing susceptibility to anxiety and depression (10). Conversely, individuals with low cognitive flexibility often display rigid thinking and a tendency toward negative cognition, which may cause emotional stagnation, hinder the rational processing of trauma, and obstruct PTG (26, 27). Therefore, cognitive flexibility plays an important role in predicting PTG.

Based on clinical observations, we propose several effective interventions to promote positive mental health among patients with severe sepsis: (1) healthcare providers can encourage patients to engage in meaningful activities within their capability during the recovery phase, helping them to rediscover their self-worth and rebuild self-confidence, thereby facilitating personal transformation; (2) establishing a robust social support system through interpersonal interaction can enhance PTG by providing emotional reinforcement; and (3) mindfulness meditation may also be beneficial, enabling patients to observe themselves and their surroundings objectively, and helping those with negative psychological patterns shift from mental rigidity to greater cognitive and emotional flexibility (28–30).

In summary, psychological resilience exerts both direct and indirect effects on PTG in patients with severe sepsis treated with CRRT, with cognitive flexibility serving as a key mediator. Clinically, the development of targeted psychological interventions is essential for improving mental health outcomes and promoting recovery. However, this study had several limitations. First, the cross-sectional design limits the ability to infer temporal or causal relationships among variables. Second, the sample was restricted to a single geographical region, which may have affected the generalizability of the findings. Future studies should employ longitudinal designs and multi-center recruitment strategies to enhance the robustness and applicability of the results, particularly across diverse patient populations with severe sepsis.

## Data Availability

The original contributions presented in the study are included in the article/supplementary material, further inquiries can be directed to the corresponding author.
